# Endotracheal Intubation Using a Direct Laryngoscope and the Protective Performances of Respirators: A Randomized Trial

**DOI:** 10.1155/2017/7565706

**Published:** 2017-04-27

**Authors:** Taeho Lim, Sanghyun Lee, Jaehoon Oh, Hyunggoo Kang, Chiwon Ahn, Yeongtak Song, Juncheol Lee, Hyungoo Shin

**Affiliations:** ^1^Department of Emergency Medicine, College of Medicine, Hanyang University, Seoul, Republic of Korea; ^2^Convergence Technology Center for Disaster Preparedness, Hanyang University, Seoul, Republic of Korea; ^3^Department of Emergency Medicine, College of Medicine, Hallym University, Seoul, Republic of Korea

## Abstract

*Purpose*. Emergency physicians are at risk for infection during invasive procedures, and the respirators can reduce this risk. This study aimed to determine whether endotracheal intubation using direct laryngoscopes affected protection performances of respirators.* Methods*. A randomized crossover study of 24 emergency physicians was performed. We performed quantitative fit tests using respirators (cup type, fold type without a valve, and fold type with a valve) before and during intubation. The primary outcome was respirators' fit factors (FF), and secondary outcomes were acceptable protection (percentage of scores above 100 FF [FF%]).* Results*. 24 pieces of data were analyzed. Compared to fold-type respirator without a valve, FF and FF% values were lower when participants wore a cup-type respirator (200 FF [200-200] versus 200 FF [102.75–200], 100% [78.61–100] versus 74.16% [36.1–98.9]; all *P* < 0.05) or fold-type respirator with a valve (200 FF [200-200] versus 142.5 FF [63.50–200], 100% [76.10–100] versus 62.50% [8.13–100]; all *P* < 0.05). There were no significant differences in intubation time and success rate according to respirator types.* Conclusions*. Motion during endotracheal intubation using direct laryngoscopes influenced the protective performance of some respirators. Therefore, emergency physicians should identify and wear respirators that provide the best personalized fit for intended tasks.

## 1. Introduction

Emergency physicians are the front-line healthcare workers who are at risk for exposure to airborne and aerosolized infectious hazards during invasive and emergent procedures, such as endotracheal intubation [1]. Various devices and guidelines have been developed and clinically used to minimize the risk of exposure. For example, an N95 filtering facepiece respirator is recommended by many healthcare organizations [2–4]. The National Institute for Occupational Safety and Health also certifies respirators according to their filter efficiencies, in order to rank the protective performances of the various N95 respirators [5, 6]. Unfortunately, emergency physicians can still be exposed to infectious hazards while wearing certified respirators if the respirator is not properly fitted on the wearer's face [7]. For example, in the Republic of Korea, healthcare providers wearing certified N95 respirators have been infected by the Middle East Respiratory Syndrome (MERS) coronavirus after performing cardiopulmonary resuscitation on an infected patient [8]. Another study revealed that inward leakage of contaminants because of an incomplete face seal was 3–5-fold more common, compared to filter penetration [9]. Furthermore, head movements can affect respirator fit, based on a change in the relative positions of the face and respirator, which can potentially cause face-seal leaks [10, 11]. Therefore, to reflect the effects of head movements on respirator fit, the Occupational Safety and Health Administration uses a standard eight-movement procedure in most respirator fit tests [11, 12].

A direct laryngoscope is used as a first-line airway device during endotracheal intubation [13, 14], and emergency physicians must approach the patient's mouth and manipulate their airway structures to expose the vocal cords. The risk of infection can increase during endotracheal intubation using a direct laryngoscope due to aerosolized hazards which was generated during coughing and serial motions during intubations [15–17]. Serial motions during intubation could affect respirator fit and increase the risk of infection [10, 11]. However, to the best of our knowledge, no studies have examined the effect of motion during endotracheal intubation on the protective performance of respirators. The present study aimed to determine whether endotracheal intubation using a direct laryngoscope affected the protection performances of various respirators.

## 2. Methods

### 2.1. Study Design

This randomized crossover study was performed at a Korean university medical center in September 2016. The local ethics committee approved this study in January 2016 (HYUH-2015-10-008-003), and all physician participants provided their informed consent for participation. No patients were involved in this study. The study protocol was registered with the Clinical Research Information Service before the study's initiation (cris.nih.go.kr: KCT0001802).

### 2.2. Participants

We recruited emergency physicians who were working at a tertiary medical center in August 2016. All participants were healthy volunteers who were 16–60 years old and had performed >50 intubations using a direct laryngoscope. We excluded subjects who had lung disease (uncontrolled chronic asthma or pneumonia), high blood pressure (>160/95 mmHg), or wrist and lower back disease. The sample size was calculated based on a pilot study of 8 participants, which examined the change in fit factor before and during intubation using a direct laryngoscope while wearing a cup-type respirator. The mean (SD) fit factors were 154.58 (37.83) before the intubation and 128.71 (41.07) during the intubation (details regarding this calculation are provided in the following section). The estimated sample size calculation (G-Power 3.1.2®; Heinrich Heine University Düsseldorf, Düsseldorf, Germany) revealed a required sample of 21 participants (effect size of 0.57, a-error of 0.05, and power of 0.8), although 24 participants were enrolled to account for a 10% dropout rate.

### 2.3. Equipment and Materials

Three types of N95 or higher respirators were used in this study: (1) cup-type respirators that are preformed into a cup shape (1860 or 1860S; 3M, Elyria, OH, USA), (2) fold-type respirators that are flexible and free-folded (1870; 3M, Elyria, OH, USA), and (3) valve-type respirators that are similar to the fold-type respirator and have a valve for reducing exhalation resistance (9332; 3M, Elyria, OH, USA). These respirators were selected because they were usually used in emergency medical centers during the South Korean MERS epidemic.

**Figure 1:**
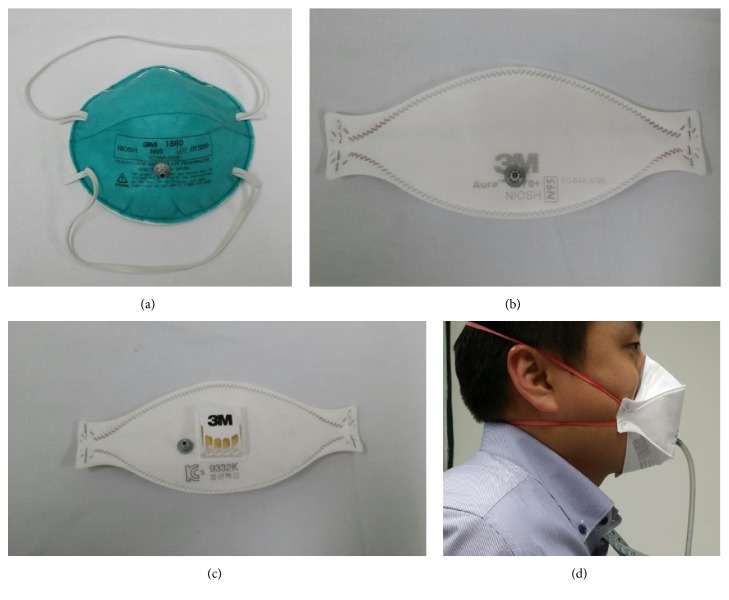
Quantitative fit test was performed using the PortaCount Plus (TSI Inc., St. Paul, MN). (a) Cup type which is preformed to cup shape (3M 1860 or 3M 1860S (small sized)). (b) Fold type which is flexible and free-folded (3M 1870). (c) Valve type which is similar to the fold type with the valve reducing the exhalation resistance (3M 9332). (d) One sampling tube was connected to the respirator and the other sampling tube was exposed to the atmosphere.

The quantitative fits of respirators were tested using the PortaCount® Plus 8038 (TSI Inc., St. Paul, MN) (Figure 1). This device is equipped with two sampling tubes, where one sampling tube is exposed to the atmosphere and measures ambient particles and the other sampling tube is connected to the respirator and measures particles in the respirator. The fit factor is calculated using the ratio of the measured ambient particles to the intrarespirator particles, with a maximum score of 200 and a score of >100 considered sufficient for passing the fit test [18]. The tubes are supported by a wire hanging around the participant's neck to support the weight of the tubes.

Intubation was performed using the Macintosh laryngoscope, which has a size 4 curved blade with a Satin Slip Stylet (Mallinckrodt Medical, St. Louis, MO), and the Airway Management Trainer (Laerdal, Stavanger, Norway). Endotracheal tubes with a standard cuff and 8.0 mm internal diameter (Lo-Contour Murphy; Mallinckrodt Medical, Athlone, Ireland) were used in this study. During the test, a manikin was placed on a bed (Transport Stretcher, 760 × 2,110 mm, 228 kg; Stryker Co., Kalamazoo, MI, USA), and the bed's height was adjusted to approximately the height of the participant's midchest level.

### 2.4. Interventions

**Figure 2:**
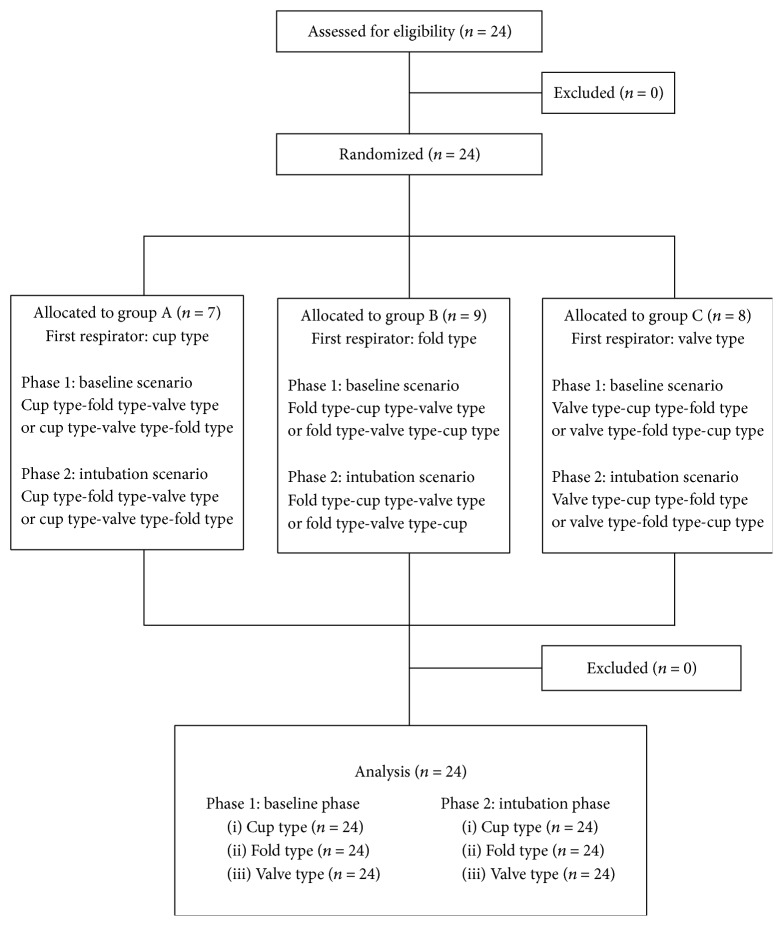
Diagram showing the flow of participants through the study.

All participants completed a brief questionnaire regarding their demographic information (age, sex, body weight, and height) and prior clinical experience with donning respirators and intubations using direct laryngoscopes. All 24 participants were randomly allocated into three groups (https://www.random.org) and assigned one of the three respirators to begin the testing (Figure 2). Each participant completed the quantitative mask fit testing using N95-companion mode for each respirator type. All participants were prohibited from smoking, eating, chewing gum, and drinking (except for plain water) for at least 30 min before starting the quantitative fit test.

The testing was performed in a resuscitation room (24.3 m^3^) without an operating air conditioning system to minimize the effect of air conditioning system on the concentration of particles. The TSI 8026 Particle Generator was used to generate a sodium chloride aerosol to ensure that the ambient air contained at least 100 particles/cc in the proper size range [19]. All participants were assigned respirators based on their face and lip length measurements, as recommended by the Los Alamos National Laboratory [20]. Before the testing, the participants were allowed to practice and learn about the respirators using manuals that were created by our Department of Infection Management. The fit factors for all three respirators were measured at two phases: (1) at baseline before the intubation and (2) during the intubation. During the baseline phase, the fit factor was measured during a period of 2 min with normal breathing after the user had sealed the respirator based on the manufacturers' instructions. During the intubation phase, the fit factor was measured from the blade's insertion into the manikin's oral cavity to the first bagging after the intubation. All participants were provided with 10-minute breaks between each test.

### 2.5. Outcomes

The primary outcomes were the fit factors for the three respirators. The secondary outcomes were the provision of adequate protection, which was defined as the percentage of fit factor scores of ≥100 (i.e., the respirator provided proper protection) [18], the physicians' respirator preferences, intubation time, and intubation success rate. The tests were timed from the point when the participant inserted the blade between the manikin's teeth after the investigator's command. The end point was at the first manual ventilation after the intubation, regardless of whether the manikin's lungs inflated. We defined intubation failure as improper tube tip placement (i.e., in the esophagus or oral cavity, rather than the trachea) or time to intubation of ≥90 s [21, 22]. The physicians' respirator preferences were recorded by asking the participant to choose a respirator that they believed provides the best protection and comfort during intubation.

### 2.6. Statistical Analysis

All data were compiled using a standard Excel spreadsheet (Microsoft, Redmond, WA, USA) and were analyzed using SPSS software (version 18.0 KO for Windows; SPSS Inc., Chicago, IL, USA). Categorical data were reported as number and percentage, while continuous data were reported as median and interquartile range (IQR) because the data were not normally distributed. The Friedman test for continuous variables was used to compare the fit factors among the three N95 respirators before and during the intubations. A post hoc analysis was performed using the Wilcoxon signed rank test and Bonferroni correction. The Wilcoxon signed rank test was used for continuous variables to compare the fit factors before and during the intubations. Differences with a *P* value of <0.05 were considered statistically significant.

## 3. Results

### 3.1. General Characteristics

All 24 participants completed the study, and their general characteristics are shown in Table 1. Eighteen participants received a Model 1860 respirator and six participants received a Model 1860S respirator, based on their face and lip measurements.

### 3.2. Fit Factors during the Baseline Phase

During the baseline phase, all three respirator types provided similar fit factor values, with a median value of 200 (*P* = 0.107). All three respirators provided adequate protection, and there were no significant differences between the three respirators (*P* = 0.081) (Table 2).

### 3.3. Fit Factors during the Intubation Phase

**Figure 3:**
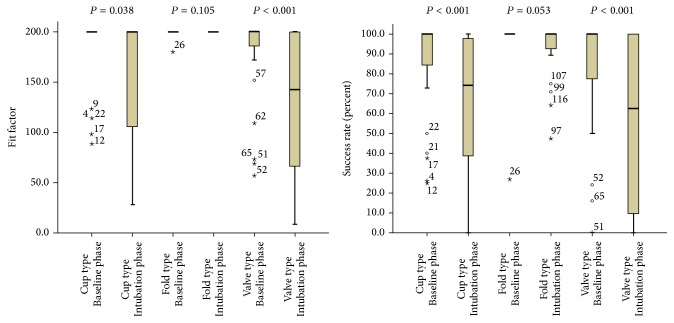
Fit factors and success rates of 3 types of N95 or higher respirators with and without intubation using direct laryngoscopes. Fit factor of valve type was decreased during intubation. Percentage of fit factor greater than or equal to 100 of cup type and valve type was decreased during intubation. ∗ means an extreme value which is more than 3 times the upper and least value. ∘ means an outlier value which is more than 3/2 times the upper and least value.

There was no significant difference when we compared the fold-type respirators before and during intubation (*P* = 0.105). However, the median fit factor for the valve-type respirators decreased during the intubation phase, compared to the baseline phase (*P* < 0.001). Both the cup-type and the valve-type respirators provided lower adequate protection rates during the intubation (both *P* < 0.001). There was a marginally significant difference in the adequate protection rates for the fold type before and during intubation using a direct laryngoscope (*P* = 0.053) (Figure 3).

### 3.4. Respirator Preferences

When asked which respirator they preferred to prevent infection during intubation, 12 participants (50.0%) preferred the valve-type respirators, 10 participants (43.5%) preferred the fold-type respirators, and 2 participants (8.7%) preferred the cup-type respirators.

### 3.5. Intubation Time and Success Rate

All three respirators had similar intubation times (*P* = 0.247), and the intubation success rates were 100% for all three respirators.

## 4. Discussion

Respirators with an N95 or higher filter have been recommended to protect physicians against infection through droplets or aerosol during invasive procedures [2–4]. However, no studies have examined the protective performances of respirators during intubation using a direct laryngoscope in the emergency room. Or et al. have demonstrated that training undergraduate nursing students to properly fit their respirators increased the likelihood of proper respirator use [23]. A component analysis of respirator user training revealed that adequate knowledge alone did not ensure proper respirator use in clinical practice [24]. In the present study, all participants received real-time feedback after their respirator training based on the manufacturer's manual. This training course could help improve the baseline respirator fit for all types of respirators.

Our simulation study revealed that motions during intubation using a direct laryngoscope could decrease the protective performance of some respirators. In this context, simulated workplace testing for respirator fit typically considers eight standard exercises: (1) normal breathing, (2) deep breathing, (3) turning the head side to side, (4) moving the head up and down, (5) talking, (6) grimacing, (7) bending over, and (8) normal breathing [11, 12]. However, bending at waist with head movement is required to expose and visualize the vocal cords during intubation using a direct laryngoscope, and we believe that these motions can decrease the protective performance of some respirators [10, 11]. Intubation using a video laryngoscope could solve this problem, by placing a camera on the blade's tip and using a display screen. Moreover, video laryngoscopes could reduce the intubation time and limit exposure, compared to direct laryngoscopes [25, 26]. In the present study, the values for adequate protection and fit factor were decreased during the intubation phase for the cup-type and valve-type respirators, but not for the fold-type respirators. These findings suggest that motion can reduce the protection from these respirators, and we believe that the user should consider the required motions when selecting and fitting a respirator for procedures that involve a risk of infection.

If a respirator's fit is disrupted, leakage can occur through three pathways: (1) filter penetration, (2) face-seal leakage, and (3) through the exhalation valve [27]. However, all filters in this study were N95 or higher, and face-seal leakage is a major component of respirator leakage [9]. In the context, fold-type and valve-type respirators have flexible sealing surfaces, while the cup type does not, which suggests that users may more easily manipulate fold-type and valve-type respirators to achieve a better face seal. However, fold-type and cup-type respirators have similar face-seal areas [19]. Interestingly, a respirator's nosepiece helps prevent face-seal leakage in the nasal area, which is the most frequently observed area [28]. In this study, the nosepiece was freely flexible in the fold-type and valve-type respirators, but not in the cup-type respirator, which may influence the difference in leakage amounts. Facepiece respirators are equipped with nonadjustable head straps for face sealing, and cup-type respirators have head straps with greater length, thickness, and cross-sectional area, compared to fold-type and valve-type respirators [19]. The different physical properties of head straps according to types of respirators could influence the respirator fit. The pressure generated by head straps could influence the respirator fit, as Niezgoda et al. found that fold-type respirators achieved similar fit factor values, compared to cup-type respirators, at the lower seal pressure that was generated by the head strap [19]. Finally, although the valve in a respirator reduces exhalation resistance [27], it also increases the risk of leakage, and additional studies are needed to quantify the leakage through the exhalation valve. Therefore, the respirator's characteristics (i.e., shape of the sealing surface, nosepiece type, head straps, and valve for reducing exhalation resistance) could influence the final respirator fit from the present study.

During outbreaks of infectious diseases and bioterrorism attacks, it may be necessary to wear respirators for prolonged periods of time. Although Rebmann et al. reported that long-term respirator use did not result in a clinically relevant physiological burden for the wearer, it was associated with many subjective symptoms [29]. Thus, wearer compliance may be linked to the selection of respirators that are comfortable and preferred by the wearer. In this context, low-pressure facial seal areas are prone to leakage, while high-pressure facial seal areas cause facial discomfort that negatively affects wearer compliance [19]. Compared to fold-type and valve-type respirators, cup-type respirators are rigid and have higher pressure that is generated by the head strap, which could increase facial discomfort and explain the low ranking for this type in our evaluation of the physician's preferences.

The changes of the respiratory rate and intensity could influence the result of respirator fit [30]. The respiratory rate and intensity could be influenced by characteristics of procedures such as workload, degree of stress, and environment. It would be needed to create a well-designed simulated workplace which reflects the changes of respiration rate and intensity for physicians who perform procedures with a high risk of infection.

## 5. Limitations

This study has several limitations. First, we used three different respirators that were used during the South Korean MERS outbreaks, with the cup-type and fold-type respirators having an N95 rating and the valve-type respirator having an FFP3 rating. Therefore, clinical trials with other respirator types are needed to confirm the effect of motion during intubation using a direct laryngoscope. Second, we performed intubation using a direct laryngoscope, and there are many different airway devices that have been developed and used clinically. Therefore, the times and motions needed to perform intubation may vary for each device, which could have different effects on respirator fit. Third, we only recruited emergency physicians from one emergency medical center, and these individuals had different levels of knowledge and experience with using respirators. Therefore, these different experiences might have influenced our findings, despite our education regarding proper respirator use before the tests. Fourth, the intubations were performed using an Airway Management Trainer (Laerdal, Stavanger, Norway), which may not reflect real-life clinical situations, despite it being a high-fidelity manikin.

## 6. Conclusions

Our findings indicate that motions during endotracheal intubation using a direct laryngoscope could influence the protective performance of some respirators. Therefore, emergency physicians should identify and wear a type of respirator that provides the best personalized fit for their intended tasks.

## Figures and Tables

**Table 1:** Demographic characteristics.

Characteristics	Data
Sex (percent)	Male, 24 (100)
Age (years)	31 (28–35)
Height (cm)	177 (171–180)
Weight (kg)	75 (69–80)
Postgraduate years (years)	4 (3–6)
Intubation experiences (times)	80 (50–100)
Face width (mm)	138.17 (10.84)
Face length (mm)	119.78 (9.59)

Categorical variables are given as numbers (percentage). Continuous variables are given as median (IQR).

**Table 2:** Fit factor of N95 or a higher mask in the baseline scenario and intubation scenario (*n* = 24).

		Types of N95 or a higher mask						
		Cup type	Fold type	Valve type	*P* value	Cup type versus fold type	Cup type versus valve type	Fold type versus valve type
		(*n* = 24)	(*n* = 24)	(*n* = 24)				
Baseline phase	Fit factor	200 (200-200)	200 (200-200)	200 (179–200)	0.107	0.015	0.225	0.009
	Adequate protection^*∗*^ (percent)	100 (78.61–100)	100 (100-100)	100 (76.1–100)	0.081	0.031	0.266	0.104
Intubation phase	Fit factor	200 (102.75–200)	200 (200-200)	142.5 (63.5–200)	<0.001	0.001	0.048	<0.001
	Adequate protection^*∗*^ (percent)	74.16 (36.1–98.91)	100 (91.61–100)	62.5 (8.13–100)	0.011	<0.001	0.118	<0.001
Intubation time	Intubation time^†^ (seconds)	33.24 (2.58)	28.69 (1.55)	29.88 (1.63)	0.247	0.324	0.70	1.00
Intubation success rate	Intubation success rate (percent)	24 (100)	24 (100)	24 (100)				
Preference	Preference	2 (8.3)	10 (41.7)	12 (50.0)				

Categorical variables are given as numbers (percentage). Continuous variables with a normal distribution are given as mean (SD). The nonparametric variables are given as median (IQR). Cup type: 3M 1860. Fold type: 3M 1870. Valve type: 3M 9332. ^*∗*^Success rate is the percentage of fit factor greater than or equal to 100. ^†^Intubation time is the time from inserting the blade into the mouth to the first ventilation.
